# Anxiety in the adult population from the onset to termination of social distancing protocols during the COVID-19: a 20-month longitudinal study

**DOI:** 10.1038/s41598-022-22686-z

**Published:** 2022-10-25

**Authors:** Asle Hoffart, Daniel J. Bauer, Sverre Urnes Johnson, Omid V.· Ebrahimi

**Affiliations:** 1 Department of Psychology, University of Oslo, Oslo, Norway; 2 Research Institute, Modum Bad Psychiatric Hospital, Postboks 33, N-3370 Vikersund, Norway; 3 Department of Psychology and Neuroscience, University of North Carolina at Chapel Hill, Chapel Hill, USA

**Keywords:** Biological techniques, Signs and symptoms

## Abstract

The social distancing protocols (SDPs) implemented as a response to the COVID-19 pandemic may seriously influence peoples’ mental health. We used a sample of 4361 Norwegian adults recruited online and stratified to be nationally representative to investigate the evolution of anxiety following each modification in national SDPs across a 20-month period from the onset of the pandemic to the reopening of society and discontinuation of SDPs. The mean anxiety level fluctuated throughout the observation period and these fluctuations were related to the stringency of the modified SDPs. Those with a high initial level almost in unison showed a substantial and lasting decrease of anxiety after the first lifting of SDPs. A sub-group of 9% had developed a persistent anxiety state during the first 3 months. Younger age, pre-existing psychiatric diagnosis, and use of unverified information platforms proved to predict marked higher anxiety in the long run. In conclusion, individuals with a high level of anxiety at the outbreak of the pandemic improved when the social distancing protocols were lifted. By contrast, a sizeable subgroup developed lasting clinical levels of anxiety during the first 3 months of the pandemic and is vulnerable to prolonged anxiety beyond the pandemic period.

## Introduction

The COVID-19 pandemic and the accompanying social distancing protocols (SDPs) have been associated with an increase in adverse mental health symptoms^1^. In particular, and not least due to the life-threatening nature of the virus, anxiety symptoms and disorders have increased^2^. A systematic review of data reporting the prevalence of anxiety disorders during the COVID-19 in 2020 estimated an additional 76.2 million (64.3 to 90.6) cases globally, an increase of 25.6% (23.2 to 28.0)^3^.

There are divergent hypotheses about the further course of anxiety beyond the pandemic outbreak. A trauma perspective on reaction to crises such as pandemics suggests that an initial short-term increase in anxiety is followed by recovery^4,5^. On the other hand, research on previous pandemics have indicated a long-term heightening of anxiety lasting beyond the pandemic^6^. Moreover, a few longitudinal studies extended into 2021 – the longest to July 2021^7^—have reported that a high anxiety level at the COVID-19 outbreak has persisted or increased^7–10^. Thus, there is a need to examine the temporal development of anxiety in the population until the pandemic is under control and the use of SDPs is terminated.

Moreover, fear of infection and general anxiety may fluctuate as related to stringent SDPs (e.g., lockdown signalling danger), by lightning and removal of SDPs (e.g., less social distancing and more danger of infection), and by reported infection rates. Accordingly, it is of importance to examine to what extent anxiety changes in consort with fluctuations in the stringency of implemented SDPs as well as in infection rates.

Furthermore, it is pertinent to study the individual development of anxiety and the extent to which the course of it varies over individuals. For instance, some individuals may reveal no change, others change from a high to constant low level or, conversely, from a low to a constant high level, with the latter individuals at a greater risk for prolonged anxiety beyond the pandemic.

Investigating predictors of these individual differences would help identify those most at risk and allow for efficient deployment of treatment resources. Studies from the early phases of the pandemic have revealed that younger age^3^, female sex^3^, lower educational level^11^, pre-existing psychiatric diagnosis^11^, being unemployed^12^, worry about job and economy^12^, use of unverified information platforms^13^, and living alone^11^ are associated with more anxiety.

To investigate the questions posed above, statistical models that make change in anxiety as outcome dependent on shifts in SDPs and addresses individual developments, that is, within-person changes are needed. This is achieved in latent change score (LCS) models as they make time-dependent *change* as opposed to time-dependent *status* the outcome of interest^14^. Moreover, these models estimate *within-person* change and thus individual differences in symptom profiles across the pandemic can be revealed. Finally, inspecting individual change profiles may reveal critical points at which individuals undergo a transition into a stable detrimental anxiety state or, conversely, from an anxiety state to a stable non-anxious state.

The purpose of the present longitudinal study of the adult Norwegian population was to investigate the evolution of anxiety following each modification of national SDPs across a 20-month period. The period lasted from the onset of the pandemic and the initial implementation of SDPs in March 2020 to the reopening of society and complete discontinuation of SDPs in September 2021. Thus, the study comprehensively investigates the evolution of anxiety from the onset to the termination of SDPs during the COVID-19 pandemic. The following research questions were investigated: (a) Does the population-level change profile of anxiety across modifications of SDPs follow the stringency of the SDPs? (b) Is there variation among individuals in initial level of and changes in anxiety across modifications of SDPs? (c) Do individuals at some point change from a non-anxious state to a stable high level of anxiety, or from an anxiety state to a stable non-anxious state? (d) How does the initial level of anxiety relate to changes from modification to modification of SDPs? (e) To what extent do the factors age, sex, education, psychiatric diagnosis, information platform preference, employment status, worry about job and economy, and living status predict initial levels of and changes in anxiety from modification to modification? (f) What is the connection between anxiety and contemporaneous COVID-19 infection rates?

## Results

### Sample characteristics and representativeness

The age of the 4361 participants ranged from 18 to 86 years (*M* = 36*.*5, *SD* = 14.8), 2,152 (49*.*6%) of them being female (compared to 49*.*5% females in the population), and 1543 (35*.*4%) having a university degree (compared to 35*.*6% in the population). The percentage of participants with preexisting psychiatric diagnosis was 19*.*0%, representative of the known rate of psychological disorders in the Norwegian adult population, which is between 16*.*7% and 25*.*0%^15^. The quota of participants sampled from each region of Norway was further proportional to each respective region size, yielding a geographically representative sample of Norway. The demographic composition of participants was stable across the 20-month period of the study, with no particular subgroup revealing disproportional attrition rates across the study period. At the final assessment, 45*.*0% of the participants were female, 38*.*4% had a university degree, 18*.*8% reported a psychiatric diagnosis, and age ranged from 18 to 85 years (*M* = 38.9, *SD* = 15.4).

### Sensitivity analyses

Sensitivity analyses were performed on the portion of participants who had provided complete data across all assessments, thus fully serving as their own controls regarding changes and fluctuations across assessments and modifications of social distancing protocols (SDPs). These analyses replicated the findings from the main sample, showing identical change profiles and predictive relationships across all analyses, with the correlation between the matrices containing the parameter estimates from this attrition-controlled sample and the main sample being *r* = 0.99.

### Model fit

Fit was excellent for the unconditional LCS model, with χ^2^ (15) = 73*.*34, RMSEA = 0.030 (90% CI 0.023 to 0.037), CFI = 0.992, TLI = 0.989, and SRMR = 0.030. The conditional LCS model also revealed good fit upon introduction of the exogenous predictors, with χ^2^(140) = 436.71, RMSEA = 0.022 (90% CI 0.020 to 0.025), CFI = 0.968, TLI = 0.952, and SRMR = 0.038. When fitting the unconditional LCS model, an improper estimate was obtained for the variance of *δη*_*t6*_. The estimated variance, though negative, was within sampling error of zero. Such *estimates* can occur even with properly specified models simply due to sampling variability^16^. We thus followed common practice and restricted the value of the parameter to zero in the final fitted model (see Table 1).

**Table 1:** The results of the unconditional and conditional latent change score (LCS) model of anxiety. *η*_*t1*_ = Latent intercept at T1 (March 2020); *δη*_*t2*_ = Latent change from T1-T2 (March – July, 2020); *δη*_*t3*_ = Latent change from T2-T3 (July – December, 2020); *δη*_*t4*_ = Latent change from T3-T4 (December 2020 – February, 2021); *δη*_*t5*_ = Latent change from T4-T5 (February – May, 2021); *δη*_*t6*_ = Latent change from T5-T6 (May – August, 2021); *δη*_*t7*_ = Latent change from T6-T7 (August—November, 2021). Age: 0 (18–30 years), 1 (31–44 years), 2 (45–64 years), 3 (65 years and above). Sex: 0 (females), 1 (males). Education level: 0 (compulsory school), 1 (upper secondary high school), 2 (student), 3 (university degree). Preexisting psychiatric diagnosis: 0 (absence), 1 (presence). Information platform preference: 1 (unmonitored information obtainment sources consisting of social media platforms such as Instagram, Snapchat, TikTok, online forums and blogs, and friends, family and peers), 0 (source-verified platforms encompassing of source-checked and recognized national, regional, and local newspapers, television, and radio channels). Employment status: 0 (unemployed), 1 (employed). Worry about job and economy: 0 (never worry about job and economy), to 12 (worries both about job and economy almost every day). Living status: 0 (not living alone), 1 (living alone). Daily COVID-19 incidence rates were retrieved from the Norwegian Public Health database of infectious disease and matched with the response date of each participant.

	**Estimate**	**SE**	**Z**	**p**
**Unconditional model**				
**1· Intercepts**				
η_t1_	5.58	0.07	76.44	< 0.001*
δη_t2_	− 0.81	0.11	− 7.54	< 0.001*
δη_t3_	0.62	0.08	8.16	< 0.001*
δη_t4_	0.03	0.07	0.41	0.681
δη_t5_	− 0.18	0.08	− 2.28	0.023*
δη_t6_	− 0.76	0.08	− 9.28	< 0.001*
δη_t7_	0.01	0.09	0.05	0.957
**2· Variances**				
η_t1_	18.58	0.51	36.14	< 0.001*
δη_t2_	28.30	0.89	31.85	< 0.001*
δη_t3_	1.76	0.28	6.27	< 0.001*
δη_t4_	0.07	0.20	0.33	0.739*
δη_t5_	1.33	0.20	6.52	< 0.001*
δη_t6_	0.00	NA	NA	NA
δη_t7_	0.65	0.30	2.20	0.028*
**3· Covariances**				
η_t1_ ~ ~ δη_t2_	− 16.34	0.60	− 27.47	< 0.001*
η_t1_ ~ ~ δη_t3_	0.20	0.26	0.75	0.451
η_t1_ ~ ~ δη_t4_	0.22	0.24	0.94	0.347
η_t1_ ~ ~ δη_t5_	− 0.59	0.27	− 2.15	0.032*
η_t1_ ~ ~ δη_t6_	− 0.95	0.28	− 3.46	< 0.001*
η_t1_ ~ ~ δη_t7_	0.05	0.30	0.15	0.879
**Conditional model**				
**1· Intercepts**				
η_t1_	5.39	0.31	17.32	< 0.001*
δη_t2_	− 1.39	0.41	− 3.36	0.001*
δη_t3_	0.15	0.39	0.38	0.705
δη_t4_	0.57	0.32	1.78	0.075
δη_t5_	− 0.07	0.40	− 0.18	0.859
δη_t6_	− 0.71	0.34	− 2.13	0.033*
δη_t7_	− 0.49	0.34	− 1.44	0.151
**2· Variances**				
η_t1_	11.13	0.37	30.26	< 0.001*
δη_t2_	22.16	0.77	28.79	< 0.001*
δη_t3_	1.71	0.28	6.07	< 0.001*
δη_t4_	0.07	0.20	0.36	0.718*
δη_t5_	1.17	0.22	5.43	< 0.001*
δη_t6_	0.43	0.24	1.78	0.075
δη_t7_	0.48	0.32	1.50	0.134
**3· Covariances**				
η_t1_ ~ ~ δη_t2_	− 9.89	0.46	− 21.25	< 0.001*
η_t1_ ~ ~ δη_t3_	0.01	0.24	0.03	0.979
η_t1_ ~ ~ δη_t4_	0.17	0.21	0.77	0.440
η_t1_ ~ ~ δη_t5_	− 0.34	0.24	− 1.42	0.157
η_t1_ ~ ~ δη_t6_	− 0.95	0.27	− 3.58	< 0.001*
η_t1_ ~ ~ δη_t7_	− 0.16	0.27	− 0.58	0.561
**4· Regression estimates**				
**4·1· Predictors of η**_**t1**_				
Age	− 0.72	0.08	− 9.62	< 0.001*
Sex	− 1.31	0.14	− 9.42	< 0.001*
Education	− 0.18	0.06	− 2.80	0.005*
Psychiatric diagnosis	4.04	0.17	23.91	< 0.001*
Info. platform preference	0.15	0.25	0.58	0.560
Employment status	− 0.89	0.17	− 5.23	< 0.001*
Worry job and economy	0.74	0.04	20.58	< 0.001*
Living status	− 0.23	0.21	− 1.10	0.273
Daily infection rate	0.28	0.10	2.80	0.005*
**4·2· Predictors of δη**_**t2**_				
Age	0.40	0.12	3.38	0.001*
Sex	0.96	0.23	4.25	< 0.001*
Education	0.28	0.11	2.60	0.009*
Psychiatric diagnosis	− 3.69	0.28	− 13.27	< 0.001*
Info. platform preference	0.58	0.36	1.61	0.107
Employment status	0.29	0.28	1.02	0.307
Worry job and economy	− 0.64	0.06	− 10.65	< 0.001*
Living status	0.68	0.30	2.28	0.023*
Daily infection rate	2.84	1.10	2.58	0.010*
**4·3· Predictors of δη**_**t3**_				
Age	0.10	0.09	1.16	0.246
Sex	− 0.12	0.17	− 0.68	0.495
Education	− 0.11	0.08	− 1.34	0.179
Psychiatric diagnosis	0.34	0.21	1.63	0.104
Info. platform preference	− 0.01	0.24	− 0.04	0.966
Employment status	0.45	0.21	2.19	0.029*
Worry job and economy	− 0.00	0.05	− 0.03	0.980
Living status	0.15	0.20	0.79	0.429
Daily infection rate	0.03	0.06	0.60	0.546
**4·4· Predictors of δη**_**t4**_				
Age	− 0.03	0.08	− 0.37	0.711
Sex	0.01	0.16	0.06	0.953
Education	− 0.05	0.08	− 0.64	0.523
Psychiatric diagnosis	0.07	0.20	0.34	0.734
Info. platform preference	0.04	0.25	0.14	0.887
Employment status	− 0.02	0.20	− 0.10	0.925
Worry job and economy	− 0.07	0.04	− 1.65	0.100
Living status	− 0.41	0.20	− 2.06	0.040*
Daily infection rate	− 0.11	0.08	− 1.32	0.186
**4·5· Predictors of δη**_**t5**_				
Age	0.04	0.09	0.47	0.640
Sex	0.11	0.18	0.61	0.544
Education	0.01	0.08	0.10	0.924
Psychiatric diagnosis	0.13	0.22	0.60	0.548
Info. platform preference	− 0.09	0.27	− 0.32	0.750
Employment status	− 0.04	0.22	− 0.20	0.844
Worry job and economy	0.06	0.05	1.29	0.198
Living status	0.06	0.21	0.26	0.791
Daily infection rate	− 0.06	0.08	− 0.74	0.457
**4·6· Predictors of δη**_**t6**_				
Age	0.02	0.10	0.20	0.845
Sex	0.08	0.19	0.45	0.650
Education	− 0.07	0.09	− 0.74	0.459
Psychiatric diagnosis	− 0.04	0.24	− 0.19	0.851
Info. platform preference	− 0.22	0.28	− 0.78	0.437
Employment status	0.17	0.23	0.74	0.462
Worry job and economy	0.04	0.05	0.80	0.426
Living status	− 0.15	0.22	− 0.71	0.479
Daily infection rate	− 0.10	0.10	− 1.01	0.313
**4·7· Predictors of δη**_**t7**_				
Age	− 0.13	0.10	− 1.26	0.205
Sex	− 0.04	0.20	− 0.18	0.858
Education	0.20	0.09	2.17	0.030*
Psychiatric diagnosis	0.20	0.25	0.81	0.419
Info. platform preference	0.73	0.30	2.40	0.016*
Employment status	− 0.12	0.24	− 0.50	0.618
Worry job and economy	− 0.04	0.05	− 0.84	0.402
Living status	0.08	0.23	0.36	0.722
Daily infection rate	0.03	0.01	2.10	0.036*

### Group-level anxiety profile across the pandemic period

**Figure 1:**
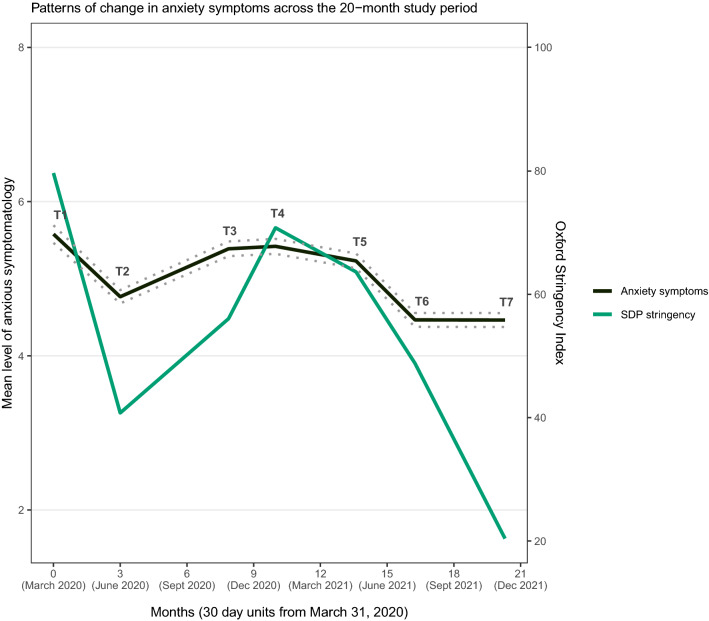
The mean-level profile of anxiety and the strictness of social distancing protocols (SDPs) over the 20-month observation period. Anxiety change patterns were modelled upon all modifications in national SDPs over the pandemic period. The dashed lines represent the 95% confidence intervals. Each month is coded in units of 30 days ensuing the starting point March 31, 2020, coded as 0.

Figure 1 displays the mean-level profile of anxiety over the observation period, with each breaking point in the curve representing an assessment interval. The strictness of the SDPs at the intervals is also displayed. From the introduction of SDPs (T1) to their discontinuation (T7), the latent anxiety level changed from 5.6 (*SD* = 3.8) to 4.5 (*SD* = 3.1). Across the five in-between modifications, anxiety severity fluctuated between these levels. Between adjacent time points, latent anxiety decreased from T1 and T2, increased from T2 to T3, and decreased from T4 to T5 and from T5 to T6 (see intercepts in Table 1). Thus, anxiety co-varied with the strictness of SDPs, with increases and decreases in strictness being associated with subsequent increases and decreases in anxiety, respectively. One exception from the overall pattern occurred from T6 to T7, where no notable anxiety change was observed despite that SDPs were discontinued. The correlation over the observation period between anxiety and strictness was 0.88 and between anxiety and mean daily infection rate in the measurement intervals was − 0.24.

### Individual variation of anxiety profiles

**Figure 2:**
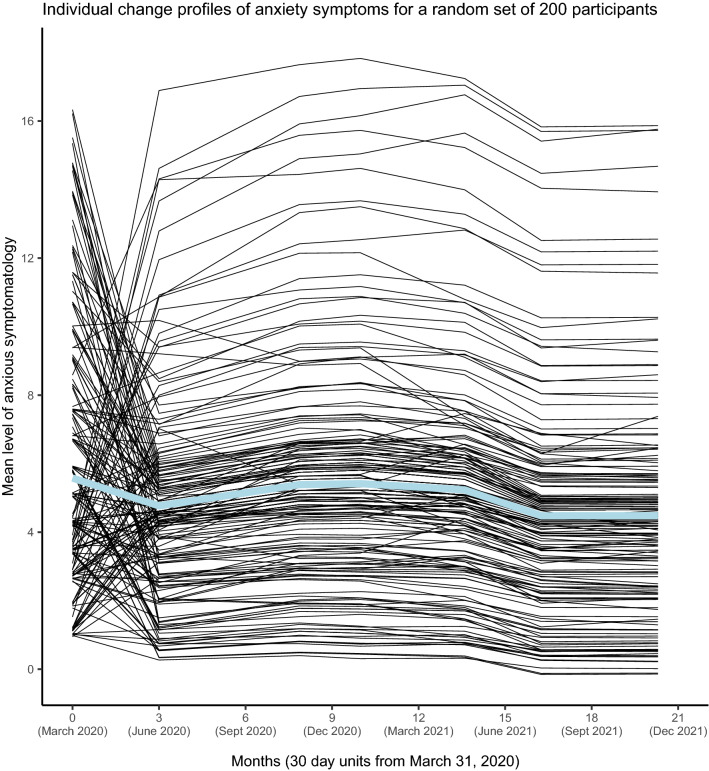
Individual change profiles in anxiety across modifications in social distancing protocols (SDPs). Through a 20-month period from the introduction of SDPs to their discontinuation.

The population average anxiety profile and the individual change profiles across the 20-month study period are exhibited in Fig. 2. For visualization purposes, the change profiles of a random subset of 200 individuals are displayed, representative of the sample. Individual change profiles of *all* participants in the study are presented in segments of 400 through 11 subfigures in online Supplementary Fig. S1.

Most variation in change profiles occurred within the first three months from T1 to T2 (Table 1). The large variation of T2 change scores is reflected in the major presence of intersecting lines between T1 and T2 in Fig. 2. The figure shows that those with a high initial anxiety level almost in unison experienced decreases, many of them considerably. Indeed, the correlation between T1 status and change from T1 to T2 was − 0.62. This negative correlation also reflects that individuals with lower initial levels of anxiety often experienced increases in anxiety from T1 to T2. These increases in anxiety often rose to a clinical level (> = 8), a level that was then prevailingly maintained across the remainder of the pandemic period. Of the 4361 participants, 394 (9.0%) had a clinically important deterioration (increase of minimal 4 points) in anxiety from T1 to T2. Notably, almost all individuals with a stable clinical level of anxiety from T2 to T7 belonged to this sub-group.

The covariances in the unconditional model (Table 1) show that a higher level of anxiety at T1 was related to more reduction of anxiety from T1 to T2, from T4 to T5, and from T5 to T6. These three periods were all associated with reduced strictness of SDPs.

### Predictors of anxiety profiles

**Figure 3:**
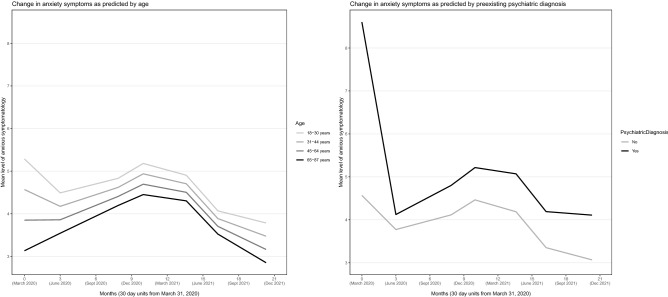
Anxiety across the 20-month observation period as predicted by age and preexisting psychiatric diagnosis. Controlled for the influence of all other variables in the model.

**Figure 4:**
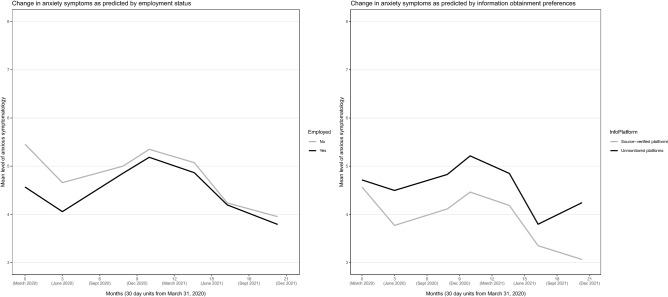
Anxiety across the 20-month observation period as predicted by employment status and information obtainment. Controlled for the influence of all other variables in the model.

**Figure 5:**
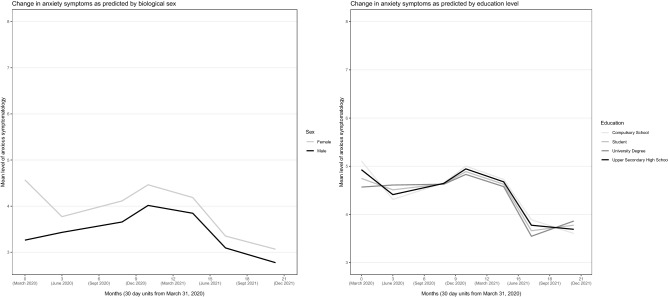
Anxiety across the 20-month observation period as predicted by biological sex and education level. Controlled for the influence of all other variables in the model.

**Figure 6:**
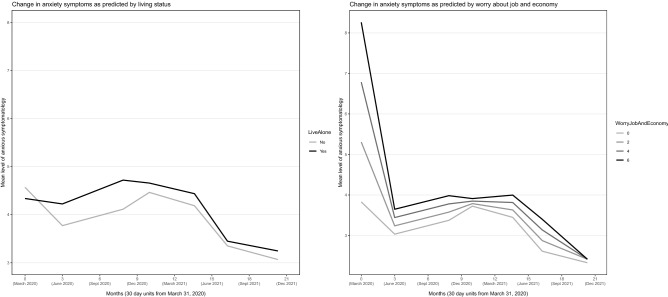
Anxiety across the 20-month observation period as predicted by living status and worry about job and economy. Controlled for the influence of all other variables in the model.

The effect of each of the exogenous predictors on the initial level and the time-point to time-point changes in anxiety, while controlling for all other predictors in the model, is reported in Table 1. These effects are also displayed in Figs. 3, 4, 5 and 6.

Younger age, female sex, lower education, pre-existing psychiatric diagnosis, unemployment, and more worry about job and economy were related to higher initial level of anxiety (i.e., *η*_*t1*_, *P*-values < 0.05), but these variable values (except unemployment) were also related to more *reductions* in anxiety from T1 to T2 (Table 1). Younger age and pre-existing psychiatric diagnosis were related to marked higher levels of anxiety over the whole observation period (Fig. 3). Preference for unverified information platforms was related to markedly higher anxiety levels from T2 and onward (Fig. 4), and to a significantly larger anxiety increase from T6 to T7. Females, unemployed, and those living alone had a somewhat higher anxiety level than their counterparts over the observation period (Figs. 5 and 6). Educational level did not influence anxiety beyond baseline, except that those with a university degree had the largest increase from T6 to T7 (Fig. 5). Worry about job and economy had a variable influence with no relationship to the end (T7) level of anxiety (Fig. 6). More worry was associated with more increase of anxiety from T3 to T4. Daily infection rate was positively related to anxiety at T1, to anxiety change from T1 to T2, and to anxiety change from T6 to T7.

## Discussion

The present results demonstrated that the mean anxiety severity fluctuated as a function of the stringency and leniency of SDPs, with increased stringency being associated with heightened anxiety. An exception from this general pattern was that there were no signs of anxiety reduction ensuing the complete discontinuation of SDPs. However, although the message from the government was that the pandemic was under control, the mean infection rate was as high as 933 new cases per day and raised from 424 to 1762 during the last measurement window. This may have prevented anxiety from reducing. Overall, anxiety level correlated strongly with strictness of SDPs over the pandemic, but negligibly with infection rate.

The preponderance of variation in changes occurred from the initial implementation of SDPs (T1) to their partial discontinuation three months later (T2). Inspection of individual change profiles revealed two change patterns consistent with critical transitions to a new stable state^17^. Individuals who had an initial high level of anxiety – above or around the clinical cut-off of 8 – almost in unison showed a substantial and lasting decrease of anxiety after the SDPs had been lifted. This is consistent with a trauma perspective on reactions to the pandemic^4^ and may reflect that they after an initial increase of anxiety somehow adapted to the stressors posed by the infection pressure and the SDPs. They remained in a non-anxious state and were predominantly resilient toward new infection waves and re-implementations of strict SDPs. A second change pattern consistent with critical transitions was seen for individuals with initially low anxiety who experienced a clinically important increase to a clinical level, which then was maintained throughout the pandemic to the discontinuation of SDPs. This critical increase was situated within the first three months of the pandemic and may have occurred before and/or after the modification of SDPs. In the former case, the individual experienced stressors of a severity that overloaded their resilience and access to environmental resources. The lifting of the SDPs seemed to have little mitigating effect. In the latter case, the anxiety increase may be a response to the SDPs discontinuation. The discontinuation may have led to heightened degree of worry about contagion or increased social anxiety. In any case, these individuals remained in a chronic anxiety state and are vulnerable to prolonged anxiety beyond the pandemic.

Consistent with previous studies, younger age^3^, female sex^3^, lower education^11^, pre-existing psychiatric diagnosis^11^, unemployment^12^, and more worry about job and economy^12^ were significantly associated with higher initial levels of anxiety. On the other hand, the same predictors were related to *greater* reductions in anxiety from T1 to T2, probably reflecting that those sub-groups most vulnerable to negative influence of infection rates and SDPs also were more relieved when the infections rates decreased to a minimum and the SDPs were partly discontinued.

Younger age, pre-existing psychiatric diagnosis, and use of unverified information platforms predicted higher anxiety in the long run. The lives of young people involve more social contact and activities than those of older people and younger people may therefore suffer more from the SDPs and the lockdown^18^. Unverified platforms may spread exaggerated or false information about the dangers of the pandemic and thus lead to heightened anxiety^13^. It is reasonable that people with a pre-existing psychiatric diagnosis also are more vulnerable to heightened anxiety later. Notably, use of unverified platforms and psychiatric diagnosis did not predict end level for depression in the same sample^19^.

Also females, unemployed, and those living alone exhibited a heightened overall anxiety level, but differences were less marked. Higher educational level was related to lower initial level of anxiety but predicted more increase of anxiety at reopening (T7). Those with a university degree and students may have been exposed to a larger increase of perceived dangers (e.g., social threats, physical closeness) as a result of the discontinuation of SDPs. Worry about job and economy at the introduction of SDPs (T1) had an impact on anxiety in the early phase of the pandemic but had no relationship to the end (T7) level of anxiety.

Daily infection rate was related to anxiety early in the pandemic, probably reflecting that there existed more uncertainty about the dangerous consequences of the virus at this stage. In addition, infection rate was related to anxiety change from T6 to T7. This may reflect the steep increase of infected cases after society had been re-opened.

Some important variables were not included the present study. For instance, sleep quality and physical activity have both been found to have decreased during COVID-19 related home confinement^20^, and both variables have been found to be associated with mental wellbeing during confinement^21^, Also during confinement, lower physical activity have been found to be related to poorer sleep^22,23^ and more anxiety^23^. Thus, physical activity and sleep quality could both contribute to the prediction of anxiety profiles, both independently and in overlap with the studied predictors (e.g., sleep and worry about job and economy).

A major strength of this study was that anxiety was measured at every modification of SDPs until the end of the pandemic containment policies and the proclaimed end of the pandemic. The pandemic was said to be under control and people could return to a normal everyday life. No knowledge of the new omicron variant and the associated infection wave was available during the last measurement window. Thus, the findings represent information about anxiety reactions from the start to what was at the time the perceived end of a pandemic. Other strengths include the large and representative sample, the simultaneous unveiling of population-level and individual change profiles, the state-of-the-art approach to missing data, and the sensitivity analyses on an attrition-controlled sample.

Some limitations should be noted. Although the participants were randomly obtained from a larger sample and stratified to represent population demographics, the initial recruitment through an online procedure may favor particular sub-groups (e.g., younger people) and involve self-selection biases. Efforts were taken to reduce such biases through additional recruitment of participants across a variety of platforms more accessible to the elderly population. The use of self-reports precluded diagnostic assessment of the participants. Potential predictors such as sleep and physical activity were not included.

In conclusion, the mean anxiety level fluctuated throughout the observation period and these fluctuations were positively related to the stringency of the modified SDPs. A sub-group of 9% who developed a chronic anxiety state during the first three months of the pandemic was identified. This sizable subgroup maintained their heightened anxiety level throughout the pandemic and is vulnerable to prolonged anxiety beyond the pandemic period. Therefore, efforts to mitigate detrimental anxiety symptomatology should focus on the early phase of pandemics and future research should identify the particular circumstances and psychological processes leading to and maintaining this chronic anxiety state. Among variables shown in other studies to predict initial anxiety response to the COVID-19 pandemic and associated SDPs, younger age, pre-existing psychiatric diagnosis, and use of unverified information platforms proved to markedly predict higher anxiety in the long run. Measures should be taken to stimulate people to use verified information platforms about the pandemic.

## Methods

The study was ethically approved by The Regional Committee for Medical and Health Research Ethics South East Norway (reference: 125,510) and the Norwegian Centre for Research Data (reference: 802,810). The study was pre-registered prior to collection of data at Clinicaltrials.gov (Identifier: NCT04442204) and conducted in accordance with the guidelines of the Strengthening the Reporting of Observational Studies in Epidemiology statement (STROBE)^24^. Digital informed consent was obtained from all participants before completing the questionnaire.

### Study design

The study period lasted 20 months from the onset of the pandemic and the introduction of national SDPs in Norway to their complete discontinuation. The design criteria included a) measuring anxiety following *each* modification of SDPs, b) initiating measurements in a two-week interval between two to four weeks following the modification, and c) stopping data collection instantaneously if novel information was provided concerning forthcoming modifications of SDPs to control for expectations effects. Hence, the timing of the measurements was based on the timing of the implementation of SDPs.

### Population, recruitment, and procedure

The targeted population for the present study was adults (age >  = 18 years) living in Norway across the period of assessment. The majority of the sample (70%) was obtained using a Facebook Business algorithm, which proportionally targeted each geographic region according to its relative size (see flowchart in online Supplementary Fig. S2). The 15% adults not present on Facebook were recruited through a systematic dissemination of the survey via national, regional, and local information platforms (i.e., television, radio, and newspapers). This procedure is explained in detail elsewhere^25^. A total of 10,061 adults enrolled in the study at T1. The same participants were recontacted at each assessment. The number participants responding at each assessment was 4,967 at T2, 5,283 at T3, 4,607 at T4, 4,228 at T5, 3,231 at T6, and 3,330 at T7.

### Stratification of sample

Characteristics not fully representative of the Norwegian adult population were post-stratified to be proportional to their known rate in the general adult population, matching each parameter in the sample to the population parameter to provide a representative sample of the Norwegian adult population. The final stratified and representative sample used in this study consisted of 4,361 of the 10,061 adults, selecting 4361 at T1, 2,151 at T2, 2239 at T3, 1963 at T4, 1811 at T5, 1,405 at T6, and 1426 at T7.

### Assessment intervals, modifications in SDPs and infection pressure

A comprehensive list of nationally implemented SDPs at each assessment interval (i.e., T1–T6) is presented in Supplementary Tables S1–S6. At T7, national SDPs were discontinued. The intervals, the strictness of the SDPs measured by the Oxford COVID-19 Stringency Index^26^, the date of their implementation, and the mean daily infection rate in the intervals, retrieved from the Norwegian Public Health database of infectious disease, were as follows:

T1 (March 31 to April 7, 2020). *Strict* SDPs: 79.63, implemented from March 13. The infection rate was 191 (*SD* = 55).

T2 (June to July 13, 2020). *Lenient* SDPs: 40.74, implemented from June 15. The infection rate was 20 (*SD* = 8).

T3 (November 19 to December 2, 2020). *Strict* SDPs: 56.02, implemented from October 26. The infection rate was 517 (*SD* = 11).

T4 (January 23 to February 2, 2021). *Increased strictness of* SDPs: 70.76, including stronger restrictions on social contact than in T1 and T3, implemented from January 4. The infection rate was 249 (*SD* = 67).

T5 (May 8 to May 25, 2021). *Decreased strictness of* SDPs: 63.61, implemented from April 16. The infection rate was 373 (*SD* = 76).

T6 (July 4 to August 1, 2021). *Lenient* SDPs: 48.79, consisting of minor distancing protocols, implemented from June 18. The infection rate was 164 (*SD* = 69).

T7 (October 24 to November 12, 2021). All SDPs were discontinued from September 24. A vaccine rate of 77% had been reached and the government declared that the pandemic was under control and that people could resume normal life. The infection rate was 933 (*SD* = 508).

### Measurement

Strictness of the national SDPs was measured by the Oxford COVID-19 Stringency Index^26^, which is based on nine metrics, yielding a final strictness score ranging from 0 (no protocols present) to 100 (strictest response possible). The nine metrics include: 1) workplace closures; 2) school closures; 3) cancellation of public events; 4) closures of public transport; 5) stay-at-home requirements; 6) restrictions on public gatherings; 7) public information campaigns; 8) restrictions on internal movements; and 9) international travel controls.

The participants reported their age, biological sex, education level, presence of preexisting psychiatric diagnosis, preferred platform for obtaining information about the pandemic and its mitigation protocols, employment status, worry about job and economy, and living status. The age of the participants was coded into four categories (i.e., 0: 18–30 years; 1: 31–44 years; 2: 45–64 years; and 3: 65 years and above). Females were coded as 0 and males as 1. Education level consisted of four categories (i.e., 0: Compulsory School; 1: Upper Secondary High School; 2: Student; 3: Any University Degree). The presence of preexisting psychiatric diagnosis was coded as 1 and its absence as 0. Use of source-verified platforms encompassing source-checked and recognized national, regional, and local newspapers, television, and radio channels was coded as 0: Source-verified information platform preference; while use of unmonitored information obtainment sources consisting of social media platforms (e.g., Instagram, Snapchat, TikTok), online forums and blogs, and friends, family and peers were coded as 1: Unmonitored information platform preference; Worry about job and economy was measured on a scale from 0: Never worry about job and economy to 12: worries both about job and economy almost every day. Living status was coded 0: Not living alone and 1: Living alone. Daily COVID-19 incidence rates were retrieved from the Norwegian Public Health database of infectious disease and matched with the response date of each participant.

The Generalized Anxiety Disorder-7 (GAD-7)^27^ consists of seven items covering the DSM-IV symptom criteria for GAD. Subjects are asked for the presence of symptoms during the past two weeks. The items are scored on a four-point scale ranging from 0 (not at all) to 3 (almost every day). The total score ranges from 0 to 21. The GAD-7 has revealed construct validity and reliability^27,28^ and has been formally translated to Norwegian^28^. As cut-offs were >  = 8 used for clinical level^15^ and >  = 4 for clinically important change^29^. The internal consistency was excellent in this sample, with Cronbach’s α ranging from 0.88 to 0.91 across assessments.

### Statistical analyses

The statistical analyses of this study were performed using R^30^. A Latent Change Score (LCS)^14^ model was used to model the development of anxious symptomatology across the 20-month study period. It was specified using the ‘lavaan’ package^31^in R. As the LCS framework concerns within-person and time-dependent change, it is a powerful technique for modeling individual fluctuations related to modifications of SDPs across the pandemic period.

**Figure 7:**
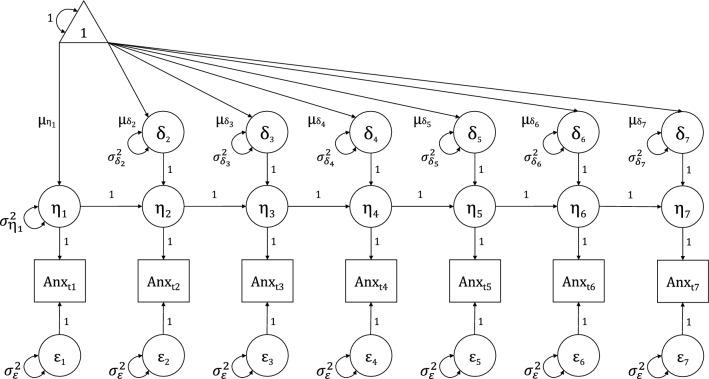
The unconditional latent change score (LCS) model. Error **v**ariances (σ^2^) are constrained to be equal. The covariances between *η*_*t*1_ and the latent change scores δη_t2−t7_ are omitted from the figure to aid visualization.

First, an unconditional LCS was fitted to the data, modeling the initial level (i.e., denoted as *η*_*t*1_) of anxious symptomatology at the first assessment interval (T1), and the latent change scores between all adjacent intervals (i.e., T1 to T2; T2 to T3; T3 to T4; T4 to T5; T5 to T6; and T6 to T7), denoted as *δη*_*t2*_*, δη*_*t3*_*, δη*_*t4*_*, δη*_*t5*_*, δη*_*t6;*_* and δη*_*t7*_, respectively (Fig. 7). T1 was coded as month 0 of the study. The residual variances (i.e., ${\sigma }_{\varepsilon }^{2}$) were held equal across assessments. Appropriate model fit was determined using common evaluation guidelines as indicated by RMSEA ≤ 0.05, TLI ≥ 0.95, CFI ≥ 0.95, and SRMR ≤ 0.05^32^. Next, the predictors and infection rate were added to yield a conditional LCS model, revealing the extent to which these variables were associated with profiles of change in anxious symptomatology across the 20-month pandemic period. Full Information Maximum Likelihood (FIML) was utilized to estimate models on the full data set, allowing for the inclusion of *individuals* with partially missing data^33,34^.

## Data availability

The data that support the findings of this study are available from the Norwegian Centre for Research Data but restrictions apply to the availability of these data, which were used under license for the current study, and so are not publicly available. Data are however available from the authors upon reasonable request and with permission of the Regional Committee for Medical and Health Research Ethics South East Norway and the Norwegian Centre for Research Data.

## Supplementary Information

Supplementary Information.
