# Supplementary material for: Anxiety in the adult population from the onset to termination of social distancing protocols during the COVID-19: a 20-month longitudinal study
Source: Sci Rep. 2022 Oct 25;12:17846. doi: 10.1038/s41598-022-22686-z (PMC9593999; doi:10.1038/s41598-022-22686-z)
Supplement: Supplementary file 1 — Supplementary Information. [file 41598_2022_22686_MOESM1_ESM.docx]

Supplementary Materials for

**Anxiety in the adult population from the onset to termination of social distancing protocols during the COVID-19: A 20-month longitudinal study**

Asle Hoffart*, Daniel J. Bauer, Sverre Urnes Johnson, Omid V. Ebrahimi

*Corresponding author. Email: [asle.hoffart@modum-bad.no](mailto:asle.hoffart@modum-bad.no)

**Content:**

Figure S1

Figure S2

Tables S1 to S6

**Figure S1.** The individual change profiles of anxiety of all participants

throughout the pandemic period in segments of 400 subjects across 11 subfigures

Through a 20-month period from the introduction of VMPs to their discontinuation.

**Figure S2**. Sampling of participants

| The adult population of Norway  (*N* = 4.2 million)  85% of the adult Norwegian population (*n* = 3.6 million) reachable through Facebook and randomly reached out to through Facebook Business algorithm  Residual 15%  (*n* = 600 000)  Reached out through:  - National Television with *n* = 1.1 million viewers at time of broadcast  - National radio stations  - Regional and local radio stations across the country  - National newspapers  - Regional and local newspapers across the country  - Additional local and regional media and social media sources  Dissemination of online survey to a random selection of 174 885 of Norwegian adults using Facebook Business algorithm  3108 respondents  6976 respondents  Total participant pool  (*n* = 10084)  Stratified sample: T1  (*n* = 4361)  T3  (*n* = 2239)  T4  (*n* = 1963)  T5  (*n* = 1811)  T6  (*n* = 1405)  T7  (*n* = 1426)  T2  (*n* = 2151)  **Table S1**. All nationally initiated social distancing protocols (SDPs) actively in place in Norway during the first period of data collection (T1; between March 31 to April 7, 2020) | | |
| --- | --- | --- |
| **SDPs** | **Duration of SDPs** | **Modification or novel information about modification of SDPs provided during the measurement period** |
| 1. Individuals who have been in contact with anyone who has been infected by the disease are quarantined for 14 days following initial contact with the infected person. | Two weeks prior to and during the measurement period. | No |
| 2. Anyone suspecting symptoms of the coronavirus or is confirmed to have the virus must be in isolation. | Two weeks prior to and during the measurement period. | No |
| 3. Social and physical distancing: individuals are disallowed from being in groups with more than five people and must maintain at least two meters distance from others. | Two weeks prior to and during the measurement period. | No |
| 4. Closing schools, kindergartens, and universities. | Two weeks prior to and during the measurement period. | No |
| 5. Close of all businesses in the catering, food, and beverage industry. The exception of the rule involves eateries that may facilitate visitors to have at least a one-meter distance from each other. | Two weeks prior to and during the measurement period. | No |
| 6. Closure of all additional businesses with increased risk of infectious spread. This includes any business involving human contact, with the exception of essential stores (e.g., grocery stores, pharmacies). | Two weeks prior to and during the measurement period. | No |
| 7. Individuals returning to Norway receive an automatic quarantine duration of 14 days. | Two weeks prior to and during the measurement period. | No |
| 8. Cancellation of cultural events (e.g., concerts), closing of gyms and physical work-out centers. | Two weeks prior to and during the measurement period. | No |
| 9. Health personnel disallowed from leaving the country. | Two weeks prior to and during the measurement period. | No |
| 10. All hospitals and health institutions must introduce access control and stop regular visitation routines. | Two weeks prior to and during the measurement period. | No |
| 11. Ban on traveling to and staying overnight at one’s leisure property outside the individuals residing municipality. | Two weeks prior to and during the measurement period. | No |
| 12. Border control: The borders are closed with regards to visitors from other countries. | Two weeks prior to and during the measurement period. | No |

| **Table S2.** All nationally initiated social distancing protocols (SDPs) actively in place in Norway during the second wave of data collection (T2; between June 22 to July 13, 2020) | | | |
| --- | --- | --- | --- |
| **SDPs** | **Duration of SDPs** | **Specific modification conducted since onset of the pandemic (T1 measurement)** | **Modification or novel information about modification of SDPs provided during the measurement period** |
| 1. Individuals who have been in contact with anyone who has been infected by the disease are quarantined for 10 days following initial contact with the infected person. | Since the onset of the pandemic protocols in Norway (March 12, 2020). | Quarantine period reduced from 14 to 10 days. *Modified May 7, 2020.* | No |
| 2. Anyone suspecting symptoms of the coronavirus or is confirmed to have the virus must be in isolation. | Since the onset of the pandemic protocols in Norway (March 12, 2020). | *Unmodified.* | No |
| 3. Social and physical distancing: individuals are disallowed from being in groups with more than twenty people and must maintain at least a one-meter distance from others. | Since the onset of the pandemic protocols in Norway (March 12, 2020). | Distance reduced from two meters to one meter. (*Modified May 7, 2020)* | No |
| 4. Universities and colleges are closed (Elementary and high school have re-opened) | Since the onset of the pandemic protocols in Norway (March 12, 2020). | Elementary and high schools are re-opened.  (*Modified May 7, 2020)* | No |
| 5. Individuals visiting or returning to Norway receive an automatic quarantine duration of 10 days. | Since the onset of the pandemic protocols in Norway (March 12, 2020). | Quarantine period reduced from 14 to 10 days. *(Modified May 7, 2020)* | No |
| 6. Public events must not exceed more than 200 individuals. In this case, they may be allowed if events can maintain the one-meter distance rule and meet the requirement of infection control protocols. | Introduced May 7, 2020. | Public events re-allowed given specific conditions.  *(Introduced May 7, 2020)* | No |
| 6. One-on-one health service providers (e.g., psychologists and physiotherapists) may re-open provided they meet the requirement of infection control protocols. | Introduced April 20, 2020. | Re-opening. *(Introduced May 7, 2020)* | No |
| 6. One-to-one contact services (e.g., hair salons), gyms, and the catering and beverage industry may re-open provided they meet the requirement of infection control protocols (as well as the maintenance of a one-meter distance for gyms and the catering and beverage industry). | Introduced April 27, 2020 (contact services), May 7, 2020 (gyms) and June 1, 2020 (catering and beverage industry). | Re-opening. *(Introduced respectively: 27 April, May 7, and June 1, 2020)* | No |
| 7. All hospitals and health institutions must introduce access control and stop regular visitation routines. | Since the onset of the pandemic protocols in Norway (March 12, 2020) | *Unmodified*. | No |

| **Table S3.** All nationally initiated social distancing protocols (SDPs) actively in place in Norway during the third wave of data collection (T3; November 19 to December 2, 2020) | | |
| --- | --- | --- |
| **SDPs** | **Duration of SDPs** | **Modification or novel information about modification of SDPs provided during the measurement period** |
| **Recommendations** |  |  |
| 1. Keep a distance of 1 meter between individuals. Keeping good hand hygiene. Stay at home if sick. | From two weeks prior to data collection and during the measurement period. | No |
| 2. Everyone must limit all social contact and stay at home as much as possible. There is a limit of five individuals visiting indoors (not including the household). | From two weeks prior to data collection and during the measurement period. | No |
|  |  |  |
| 3. Avoid unnecessary domestic travel. Limit unnecessary traveling inside of Norway. It is allowed to travel to leisure properties if one can travel without contact with other people. | From two weeks prior to data collection and during the measurement period. | No |
| 4. All universities and university colleges must reduce teaching and other activities that contribute to increased mobility, including pressure on public transport. | From two weeks prior to data collection and during the measurement period. | No |
| **Rules** |  |  |
| 1. There is an upper limit of 20 people at private gatherings in public places, and 50 people at events without fixed seating. If there is fixed seating indoors, there is an upper limit of 200 people. | From two weeks prior to data collection and during the measurement period. | No |
| 2. There is a national ban on serving alcohol after midnight. Restaurants with a license to sell alcohol cannot admit new guest after 22:00. | From two weeks prior to data collection and during the measurement period. | No |
| 3. There is extended restrictions for quarantine and travel to Norway, including, and not limited to, mandatory duty of quarantine and presenting certificate of negative Covid-19-test. Persons, including tourists and visitors, who do not have their own residence or employer in Norway must stay in quarantine hotels and get tested during the quarantine period. | From two weeks prior to data collection and during the measurement period. | No |
|  |  |  |
|  |  |  |

| **Table S4.** All nationally initiated social distancing protocols (SDPs) actively in place in Norway during the fourth wave of data collection (T4; January 23 to February 2, 2021) | | |
| --- | --- | --- |
| **SDPs** | **Duration of SDPs** | **Modification or novel information about modification of SDPs provided during the measurement period** |
| **Recommendations** |  |  |
| 1. Avoid hosting guests in your home. Wait 14 days to make private visits.  2. Everyone should limit social contact to the greatest extent possible. It is recommended that meetings with other people take place outdoors, and to avoid visits of more than five guests in addition to those who are already living together. | From three weeks prior to data collection and during the measurement period.  From three weeks prior to data collection and during the measurement period. | No |
| 3. It is recommended that all organised leisure activities, sporting activities, cultural events and indoor faith community gatherings be postponed.  4. Children in day-care facilities and primary schools may receive visits from members of their own cohort. | From three weeks prior to data collection and during the measurement period.  From one week prior to data collection and during the measurement period. | No |
| 5. Avoid all non-essential travel domestically and abroad. Stays in cabins with persons from the same household continue to be permitted provided they take place in accordance with all applicable local and national rules and guidelines. | From three weeks prior to data collection and during the measurement period. | Yes: *Modified: Do not travel domestically or internationally unless this is essential one week prior to data collection.* |
| 6. Working from home is recommended for everyone who has the option of doing so.  7. All teaching and planned events at universities, university colleges and vocational training schools will take place digitally.  8. All shopping centres and shops must introduce limits on the number of customers permitted inside to enable distancing and to control access to the premises. | From three weeks prior to data collection and during the measurement period.  From three weeks prior to data collection and during the measurement period.  From three weeks prior to data collection and during the measurement period. | No  Yes: *Modifed to when possible one week prior to data collection.*  No |
| 9. Children and young people under 20 years of age may train and participate in leisure activities as usual, both indoors and outdoors. They may also be exempt from the recommendation of a one-metre physical distance where this is necessary to conduct the activity.  10. Adults may participate in exercise outdoors, if it is possible to maintain sufficient physical distancing.  11. The elite tiers of sports are recommended to postpone all league matches for two weeks. | From one week prior to data collection and during the measurement period.  From one week prior to data collection and during the measurement period.  From one week prior to data collection and during the measurement period | No |
| 12. Cultural events such as performances, shows etc., as well as courses/conferences and religious and life stance ceremonies shall be postponed if they gather attendees from multiple municipalities. | From one week prior to data collection and during the measurement period. | No |
| **Rules** |  |  |
| 1. A maximum of ten individuals may attend private gatherings outside their own home, such as a birthday celebration in rented premises. If the private gathering is taking place outdoors, the limit is 20 attendees. | From one week prior to data collection and during the measurement period. | No |
| 2. There is a limit of ten individuals for indoor sporting events, cultural events, seminars, life stance community gatherings, ceremonies, etc., in addition to a limit of 200 individuals where everyone in the audience is seated in fixed seating. Up to 50 individuals are permitted to attend funerals, even if the seating is not fixed. | From three weeks prior to data collection and during the measurement period. | No |
| 3. A maximum of 200 people may attend outdoor events, while the limit is 600 people for events at which all members of the audience are seated in fixed seating. | From one week prior to data collection and during the measurement period. | No |
| 4. The ban on the serving of alcohol in licensed establishments will remains in effect. | From three weeks prior to data collection and during the measurement period. | No |

| **Table S5.** All nationally initiated social distancing protocols (SDPs) actively in place in Norway during the fifth wave of data collection (T5; May 8 to of May 25 2021) | | |
| --- | --- | --- |
| **SDPs** | **Duration of SDPs** | **Modification or novel information about modification of SDPs provided during the measurement period** |
| **Recommendations** |  |  |
| 1. Keep a distance of 1 meter between individuals. Keeping good hand hygiene. Stay at home if sick. | From three weeks prior to data collection and during the measurement period. | No |
| 2. Everyone should limit social contact. If possible, one should meet outdoors. There is a limit of five individuals visiting indoors (not including the household). There must be possible to keep a distance of 1 meter. | From three weeks prior to data collection and during the measurement period. | No |
| 3. There is advice against traveling to other countries. Limit traveling inside of Norway if it is not necessary to travel. It is allowed to travel to cabins and hotels, but one should not use public transportation. | From three weeks prior to data collection and during the measurement period. | No |
| 4. If it is possible, one should work from home. | From three weeks prior to data collection and during the measurement period. | No |
| 5. Children and young adults under 20 can train and participate in leisure activities as usual. Adults can participate in organized training in groups of 10 or smaller but must keep a distance of at least 1 meter. If training is outside, the group size can be up to 20 adults. | From three weeks prior to data collection and during the measurement period. | No |
| 6. It is allowed for students and employees to be at the campus, in reading halls, and the library. It is not permitted with bigger physical lectures. | From three weeks prior to data collection and during the measurement period. | No |
| **Rules** |  |  |
| 1. Events gathering individuals from different municipalities should be canceled or delayed. | From three weeks prior to data collection and during the measurement period. | No |
| 2. A maximum of 10 individuals in private gatherings inside locals outside of their own homes. A maximum of 20 individuals if the event is outside. | From three weeks prior to data collection and during the measurement period. | No |
| 3. A maximum of 10 individuals on inside events. A maximum of 50 individuals on sport and cultural events if the individuals are less than 20 years of age and from the same municipality. | From three weeks prior to data collection and during the measurement period. | No |
| 4. A maximum of 100 individuals on events inside when it is mandatory seating. | From three weeks prior to data collection and during the measurement period. | No |
| 5. A maximum of 200 individuals on events outside, and up to 600 (in cohorts of 200) individuals if there is mandatory seating and 2-meter distance between cohorts. | From three weeks prior to data collection and during the measurement period. | No |
| 6. Only allowed to serve alcohol accompanied with food. Serving alcohol is prohibited after 10 pm. | From three weeks prior to data collection and during the measurement period. | No |

| **Table S6 \| All nationally initiated social distancing protocols (SDPs) during the COVID-19 pandemic in Norway actively in place during the sixth wave of data collection (T6; July 4 to August 1, 2021)** | | |
| --- | --- | --- |
| **SDPs** | **Duration of SDPs** | **Modification or novel information about modification of SDPs provided during the measurement period** |
| **Recommendations** |  |  |
| 1. Stay at home if sick and get tested if advised by local health authorities. | Announced 4^th^ of July. | No |
|  |  |  |
| 2. Stay vigilant with hand hygiene  3. International travel not advised. | Announced 4^th^ of July. | No |
|  | From ten weeks prior to data collection and during the measurement period. | No |
| **Rules** |  |  |
| 1. Events gathering individuals from different municipalities should be canceled or delayed. | From ten weeks prior to data collection and during the measurement period. | No |
| 2. A maximum of 10 individuals in private gatherings inside locals outside of their own homes. A maximum of 20 individuals if the event is outside. | From ten weeks prior to data collection and during the measurement period. | No |
| 3. A maximum of 10 individuals on inside events. A maximum of 50 individuals on sport and cultural events if the individuals are less than 20 years of age and from the same municipality. | From ten weeks prior to data collection and during the measurement period. | No |
| 4. A maximum of 100 individuals on events inside when it is mandatory seating. | From ten weeks prior to data collection and during the measurement period. | No |
| 5. A maximum of 200 individuals on events outside, and up to 600 (in cohorts of 200) individuals if there is mandatory seating and 2-meter distance between cohorts. | From ten weeks prior to data collection and during the measurement period. | No |
| 6. Only allowed to serve alcohol accompanied with food. Serving alcohol is prohibited after 10 pm. | From ten weeks prior to data collection and during the measurement period. | No |
| 7. There is extended restrictions for quarantine and travel to Norway, including, and not limited to, presenting certificate of negative Covid-19-test. Persons, arriving from “Green Countries” will be able to enter the country without having to enter travel quarantine. | Announced 4^th^ of July. | Yes |
| 8. Persons with an EU mandated COVID certificate, from the following groups may enter without restrictions.  Minors travelling together with their parents who are exempt from the travel restrictions as a result of holding an EU Digital COVID Certificate.  Foreigners who are spouses/registered partners/cohabiting partners, children or step-children (whether minors or adults) of Norwegian citizens when their family lives together outside of Norway and they are travelling to visit Norway together with the Norwegian citizen, or are joining this citizen in Norway.  Foreigners who are spouses/registered partners/cohabiting partners, children or step-children of EEA citizens travelling on business when travelling to Norway together with the EEA citizen, or are joining this individual in Norway.  EEA citizens who are visiting a close relative resident in Norway or are travelling together with a Norwegian relative. | Announced 26^th^ of July | Yes |
